# Water molecular flow control with a (5,5) nanocoil switch

**DOI:** 10.1007/s11051-013-1889-6

**Published:** 2013-08-11

**Authors:** Shin-Pon Ju, Jenn-Sen Lin, Jin-Yuan Hsieh, Meng-Hsiung Weng, Ming-Chang Chen

**Affiliations:** 1 Department of Mechanical and Electro-Mechanical Engineering, National Sun Yat-sen University, Kaohsiung, 804 Taiwan, Republic of China; 2 Department of Mechanical Engineering, National United University, Miao-Li, 360 Taiwan Republic of China; 3 Department of Mechanical Engineering, Minghsin Institute of Technology, Hsin-Chu, 304 Taiwan Republic of China

**Keywords:** Nanocoil, Molecular dynamics, Water molecule, Diffusion coefficient

## Abstract

Molecular dynamics simulation was employed to investigate the diffusion behaviors of water molecules within a (5,5) carbon nanocoil (CNC) at different tensile strains, the length and coil diameter of CNC are 22 and 6.83 Ǻ, respectively. Condensed-phase, optimized molecular potentials for atomistic simulation studies were employed to model the interaction between atoms. The results show that the diffusion in the axial direction can be enhanced by the tensile strain and the water molecule flow can be blocked at a higher strain once the deformed areas appear at the higher strain. Moreover, the deformed (5,5) CNC at strain of 2.8 can recover its original structure at strain of 0, indicating that the adjustment of diffusion coefficient is repeatable by applying different strains in the axial direction.

## Introduction

Since carbon material is strongly hydrophobic to water (Müller et al. 1996; Martin and Kohli 2003), carbon nanotubes (CNTs) have attracted great interest for use in biological applications (Zhao et al. 2012; Bianco et al. 2005). Among those CNTs, carbon nanocoils (CNCs) are the most notable because of their 3D helical structure (Chen et al. 2003). CNCs possess high tensile strength and superelasticity (Lau et al. 2006; Liu et al. 2010), and thus they have great potential applications as nanomechanical and electromagnetic device components, such as nanosprings (Williams et al. 2003), oscillators (Papadakis et al. 2004), and nanoelastic memory (Changa and Park 2006). These outstanding mechanical properties could be also instrumental in developing a novel efficient functional nanofluidic switch for controlling the flow amount of molecules from one end to the other end of CNCs.

Many experimental and theoretical investigations have demonstrated that water molecules in a nanoscale environment show properties different from those of the molecules in a bulk system (Naguib et al. 2004; Rossi et al. 2004; Byl et al. 2006). Koga et al. (2001) found that structural and transport properties of water molecules are strongly affected by the nanoconfinement effect. Holt et al. (2006) reported that a high flow rate of water occurs when it crosses through CNT pore membranes with diameters less than 2 nm. Thomas et al. (2010) examined the pressure-driven water flow through CNTs of different radii. They found that the viscosity of water is not affected when the radius is larger than 10 nm. Zheng et al. (2005) employed molecular dynamics (MD) simulation to investigate the transport of water and methanol in hydrophobic single-walled nanotubes with various diameters. They found that hydrogen bonding plays a critical role for fluid transport across the pore.

The transport control of water in a confined space by different strain mechanisms has been widely theoretically investigated. Lu et al. (2012) employed MD simulations to investigate the flux of water inside a deformed CNT driven by an oscillating charge. Their results indicate that the oscillating frequency of the charge strongly influences the flux. Li et al. (2008) studied the structures of water confined in nanotubes undergoing axial tensile strain, and found a tendency in the water molecular structure to form chains. Fang et al. (2008) investigated the behavior of water across single-walled CNTs under different external forces on nanotube wall. Their results show that single-walled CNTs can be used as a nanoscale switch to control the water flux by the deformation of nanotube wall.

These studies demonstrate how MD simulation is a powerful tool for the investigation of structural behavior at an atomic level. In this study, we conduct MD simulations to study the diffusion behaviors of water molecules inside a (5,5) CNC during axial tension. Since the water molecules diffuse generally across cell membranes in very low numbers, this research investigates controlling the diffusion of ultra-low water flow between cells or membranes. In this study, we found that the flow of molecules is interrupted as tensile strain is applied between the two ends of the CNC because of deformations. This study provides the possibility of an excellent on–off switch application.

## Simulation detail

MD methods, implemented in the DISCOVER package (Rigby 2004; Accelrys Software Inc. 2012), were used to study the diffusion behavior of water molecules within the (5,5) CNC at different tensile strains.

MD is the principal tool to understand the dynamic behaviors of individual atoms and fine structure. MD simulations method is based on the classical Newtonian equations of motion (Eq. (1)) for all atoms in the system. The result shows the trajectory that specifies how the positions and velocities of the particles in the system alter with time. Hence, the property of the system or molecule can be evaluated by the average of trajectories.

1 $$ m_{i} \frac{{{\text{d}}^{2} r_{i} (t)}}{{{\text{d}}t^{2} }} = F_{i} (t) = - \frac{{\partial U(r_{i} )}}{{\partial r_{i} }}, $$

where *m*_*i*_, *r*_*i*_, and *F*_*i*_ are the mass, position vector, force vector of molecule *i*, respectively. *U*(*r*_*i*_) is the force field.

**Fig. 1:**
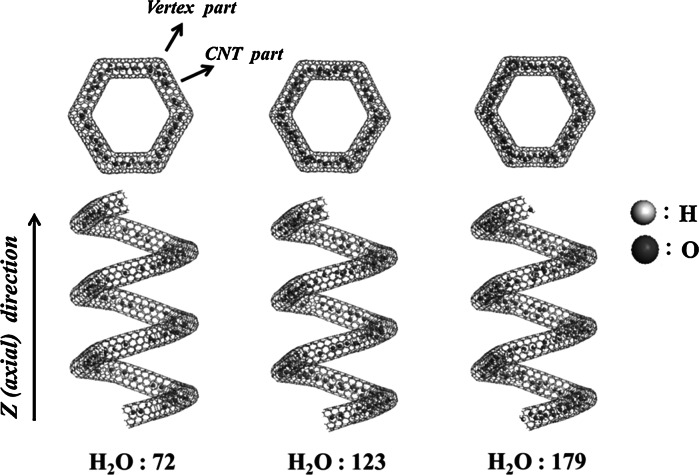
Simulation model of water molecules through the (5,5) CNC with different numbers of water molecules, 72, 123, and 179

In this simulation, the condensed-phase, optimized molecular potential for atomistic simulation studies (COMPASS) potential was employed to model the interatomic forces between the carbon atoms, between water molecules, and between water molecules and carbon atoms. The COMPASS potential *U*_total_ can be expressed as (Sun et al. 1998)

2 $$ \begin{aligned} U_{\text{total }}   = \,& U_{b} + U_{\theta } + U_{\phi } + U_{\chi } + U_{{bb^{'} }} + U_{b\theta } \\ & \quad + U_{b\phi } + U_{{\theta \theta^{'} }} + U_{{\theta \theta^{'} \phi }} + U_{\text{else}} + U_{\text{LJ}} , \\ \end{aligned} $$

3 $$ \begin{aligned} U_{\text{total}} = & \sum\limits_{b} {\left[ {k_{2} \left( {b - b_{0} } \right)^{2} + k_{3} \left( {b - b_{0} } \right)^{3} + k_{4} \left( {b - b_{0} } \right)^{4} } \right]} \\ & \quad + \sum\limits_{\theta } {\left[ {H_{2} \left( {\theta - \theta_{0} } \right)^{2} + H_{3} \left( {\theta - \theta_{0} } \right)^{3} + H_{4} \left( {\theta - \theta_{0} } \right)^{4} } \right]} \\ & \quad + \sum\limits_{\phi } {\left[ {V_{1} (1 - \cos \phi ) + V_{2} (1 - \cos 2\phi ) + V_{3} (1 - \cos 3\phi )} \right]} \\ & \quad + \sum\limits_{x} {k_{2} \chi^{2} } \\ & \quad + \sum\limits_{b} {\sum\limits_{{b^{'} }} {k_{{bb^{'} }} \left( {b - b_{0} } \right)\left( {b^{'} - b_{0}^{'} } \right)} } \\ & \quad + \sum\limits_{b} {\sum\limits_{\theta } {K_{b\theta } \left( {b - b_{0} } \right)\left( {\theta - \theta_{0} } \right)} } \\ & \quad + \sum\limits_{b} {\sum\limits_{\phi } {\left( {b - b_{0} } \right)\left[ {k_{1b\phi } \cos \phi + k_{2b\phi } \cos 2\phi + k_{3b\phi } \cos 3\phi } \right]} } \\ & \quad + \sum\limits_{\theta } {\sum\limits_{\phi } {\left( {\theta - \theta_{0} } \right)\left[ {k_{1\theta \phi } \cos \phi + k_{2\theta \phi } \cos 2\phi + k_{3\theta \phi } \cos 3\phi } \right]} } \\ & \quad + \sum\limits_{b} {\sum\limits_{{\theta^{'} }} {k_{{\theta \theta^{'} }} \left( {\theta - \theta_{0} } \right)\left( {\theta^{'} - \theta_{0}^{1} } \right)} } + \sum\limits_{\theta } {\sum\limits_{{\theta^{\prime}}} {\sum\limits_{\phi } {k_{{\theta \theta^{'} \phi }} \left( {\theta - \theta_{0} } \right)\left( {\theta^{'} - \theta_{0}^{'} } \right)\cos \phi } } } \\ & \quad + \sum\limits_{i > j} {\varepsilon_{ij} \left[ {2\left( {\frac{\sigma }{rij}} \right)^{9} - 3\left( {\frac{\sigma }{{r_{ij} }}} \right)^{6} } \right]} + \sum\limits_{i > j} {\frac{{q_{i} q_{j} }}{{r_{ij} }}} , \\ \end{aligned} $$

where potentials *U*_*b*_, *U*_*θ*_, *U*_*ϕ*_, and *U*_*χ*_ are the quartic polynomials for the bond, angle, torsion, and the out-of-plane angle coordinate, respectively. Terms 5–9 of ($$ U_{{bb^{'} }} , $$ *U*_*bθ*_, *U*_*bϕ*_, $$ U_{{\theta \theta^{'} }} , $$ and $$ U_{{\theta \theta^{'} \phi }} $$) of Eq. (2) represent the crosscoupling terms. *U*_else_ and *U*_LJ_ represent the Coulombic interaction between the atomic charges and van der Waals interactions. In this present simulation model, the water molecules were initially set inside the (5,5) CNC randomly. Figure 1 presents a simulation model of water molecules inside the periodic (5,5) CNC with different numbers of water molecules: 72, 123, and 179. The (5,5) CNC is composed of 2,580 carbon atoms, and its pitch length and coil diameter are 22 and 6.83 Ǻ, respectively. The 7-carbon and 5-carbon rings are located at the vertices of the hexagonal inner and outer walls of the (5,5) nanocoil, respectively. The segments of (5,5) CNTs are connected by these vertex portions to form a stable (5,5) nanocoils with the spiral structure. For the other CNCs, they are designated as the same indexes of the corresponding CNT segments of CNCs. In the axial tensile process, the (5,5) CNC still retains periodicity. The system temperatures are assigned a constant 300 K. The Andersen thermostat (Andersen 1980; Weinan and Li 2008) is applied to insure that the temperature of the system remains constant during simulation, and the Verlet algorithm (Verlet 1967; Swope et al. 1982) is employed to calculate the trajectories of the atoms. Moreover, all the carbon atoms of the CNC are fully relaxed during the simulation. In this study, we chose different elongated structures of strains to investigate the diffusion properties of water molecules within CNC. After CNC is strengthened, the structures are relaxed once again. The total relaxation time is set to 200 ps, because such a long equilibrium time can insure that the elongated (5,5) CNC is relaxed. The data collection and analyses were performed on the final 100 ps.

## Results and discussion

**Fig. 2:**
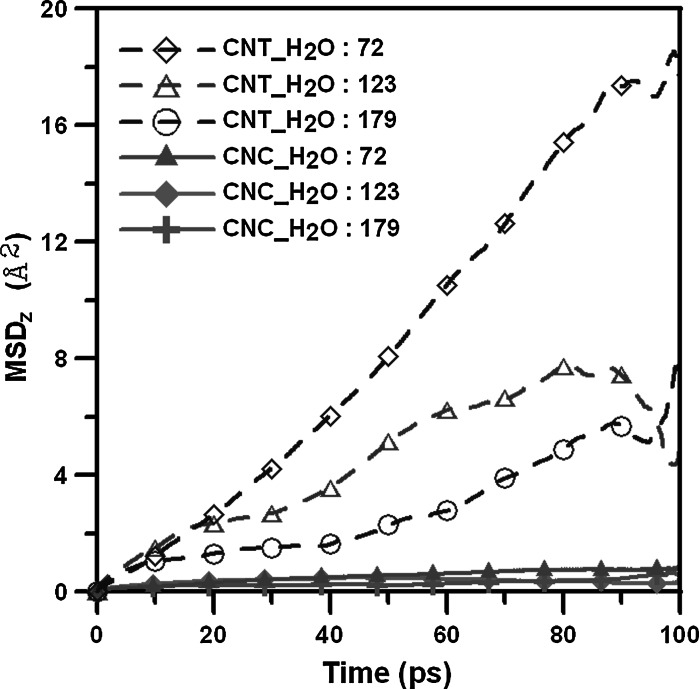
The mean-square displacement plots in the *z* dimension (MSD_*z*_) of water molecules within the (5,5) CNT and CNC at strain of 0

The *z* or axial direction is defined as the tensile direction shown in the side view of (5,5) CNC in Fig. 1, and the two different local structures are defined as the vertex and CNT parts. Figure 2 shows the mean-square displacement plots in the *z* dimension (MSD_*z*_) of water molecules within the (5,5) CNT, and CNC at strain of 0. The MSD_*z*_ is defined as

4 $$ {\text{MSD}}_{z} = \left\langle {\sum\limits_{i}^{N} {\left[ {r_{zi} (t) - r_{zi} \left( {t_{0} } \right)} \right]}^{2} /N} \right\rangle , $$

where *r*_*zi*_(*t*) represents the *z* coordinate of the oxygen atom of water molecule *i* at delay time *t*, and *r*_*zi*_(*t*_0_) indicates the referenced *z* coordinate at referenced time *t*_0_; *N* represents the total number of water molecules within CNT or CNC. The brackets are interpreted as average over time origins. The diffusion of water molecules was also examined by calculating a self-diffusion coefficient *D* in the *z* direction (*D*_*z*_) from MSD_*z*_ over the course of simulation. The self-diffusion coefficient is obtained from the MSD_*z*_ via the Einstein equation (Meunier 2005) which is rewritten as

5 $$ D_{z} = \frac{1}{2N}\mathop {\lim }\limits_{t \to \infty } \frac{\text{d}}{{{\text{d}}t}}\left\langle {\sum\limits_{i}^{N} {\left[ {r_{zi} (t) - r_{zi} (0)} \right]^{2} } } \right\rangle . $$

The diffusion coefficient of bulk water at 300 K was calculated first by Eq. (5), with the value of 2.35 × 10^−9^ m^2^/s being very close to the experimental value of 2.3 × 10^−9^ m^2^/s (Mills 1973) indicating the reliability of the COMPASS force field to predict the diffusion behavior of water molecules. In Fig. 2, MSD_*z*_ profiles of the (5,5) CNT and (5,5) CNC averaged over 100 ps (after the first 100 ps to attain equilibrium) are displayed. It shows a more significant rise in the number of water molecules within the (5,5) CNT, which indicates that water molecules have faster diffusion in the axial direction within the (5,5) CNT than within the (5,5) CNC. This is because the majority of the water diffusion direction within a (5,5) CNT is along the axial direction, and there is almost no diffusion in the plane normal to the CNT axis. For the (5,5) CNC, the MSD_*z*_ profiles are much lower than those of (5,5) CNTs, indicating the water diffusion within the (5,5) CNCs in the *z* direction is much slower than that within (5,5) CNTs. This is because water molecules within the (5,5) CNC has larger diffusion space around the *z* direction (i.e., in *x*–*y* plane).

**Fig. 3:**
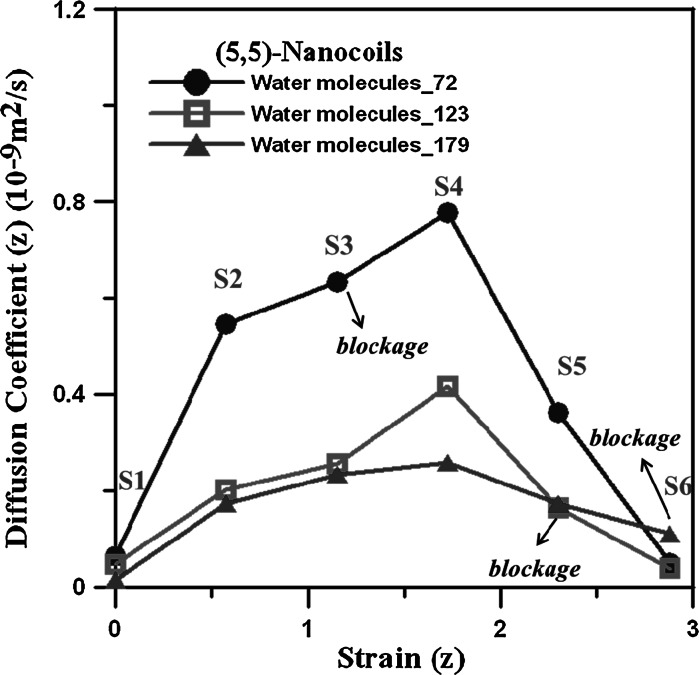
Diffusion coefficient for different water numbers within the (5,5) CNC under different strains

Since CNCs have been proven to be a superelastic material which may still be intact at a very large tensile strain (Chen et al. 2003; Liu et al. 2010), it is possible to adjust the *D*_*z*_ by applying different strains upon the (5,5) CNC. Figure 3 shows the *D*_*z*_ values for different water numbers within the (5,5) CNC. (5,5) CNCs under six different strains, labeled as S1–S6 in Fig. 3, are considered. *D*_*z*_ values first increase with the strain and then decrease after their maximal *D*_*z*_ values. Generally speaking, the diffusion within the (5,5) CNC with fewer water molecules is faster because there are fewer hydrogen bonds forming between water molecules.

**Fig. 4:**
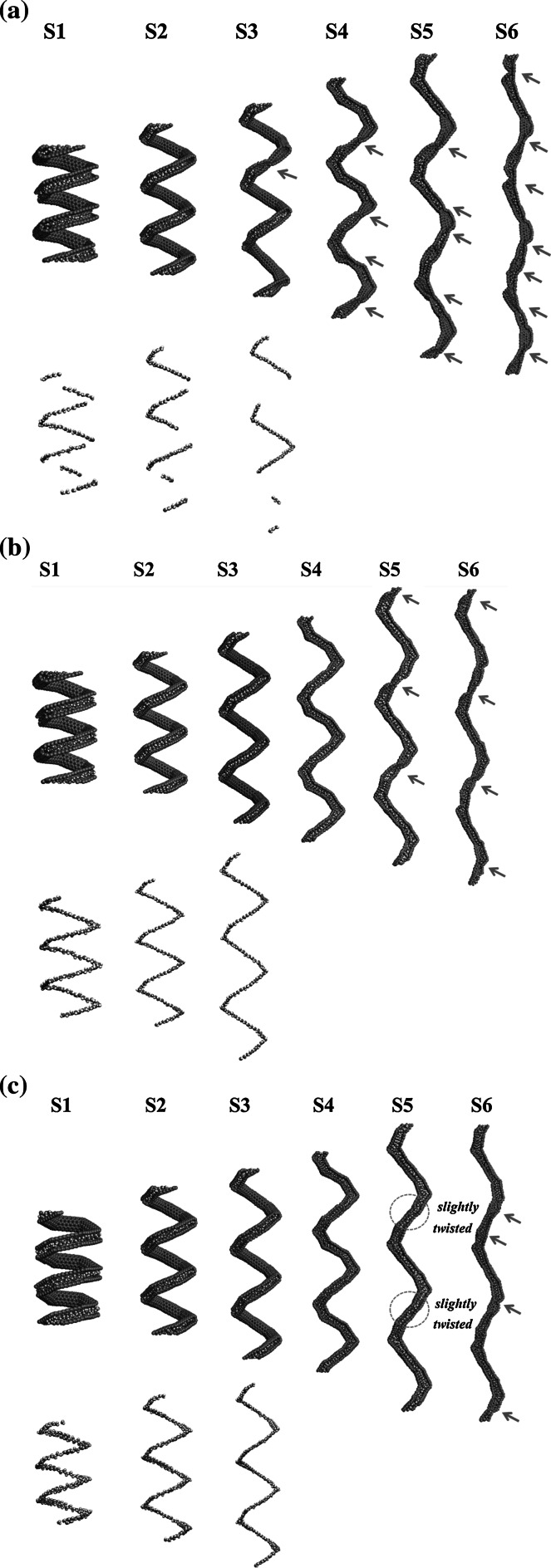
The morphologies at different strains for simulation of **a** 72, **b** 123, and **c** 179 water molecules within the (5,5) CNC

The morphologies at different strains for different water numbers within the (5,5) CNC are shown in Fig. 4a–c, and the water chain structures at the first strains are also illustrated. In Fig. 4b, the water chain at S1 indicates that 123 water molecules are sufficient to form a complete water chain within the (5,5) CNC and, for the system with 179 water molecules, it appears easier for water molecules to accumulate at the vertex parts, as shown in Fig. 4c. For the case of 123 water molecules, several shorter water chains can be seen in Fig. 4a because the water number is not sufficient, and the hydrogen bond between water molecules aids in the water molecules aggregating in a chainlike form. In Fig. 4a–c, it is clear the segments of (5,5) CNT parts are aligned more along the *z* direction with continuously increasing strain. When the strain becomes larger, some deformed areas appear, indicated by arrows in Fig. 4a–c, resulting in a blockage of water molecules within the (5,5) CNC. The strains at which the blockage occurs are at S3, S5, and S6 for cases of 72, 123, and 179 water molecules, respectively. Because the (5,5) CNC is hydrophobic, the repulsive interaction between water molecules, and the (5,5) CNC wall is relatively stronger than that between other nonpolar molecules and the CNC wall. Consequently, the higher water density within the (5,5) CNC helps in preventing deformation. In Fig. 4a, b, it is clear that more deformed areas will appear when the strain becomes larger, with the water molecules becoming distributed in different segments formed by the deformed areas. For *D*_*z*_ values in Fig. 3, the strains at which the deformations appear are indicated as “blockage” for three different water densities, indicating that the CNC channels are no longer open after those points: that is, after S3 for 72 molecules, S5 for 123 molecules, and S6 for 179 molecules. For the case of 72 water molecules, *D*_*z*_ still increases at S4 although one deformed area has appeared at S3. The reason for the diffusion coefficient of S4 being larger than S3 is because water molecules are locked locally within the segments created by deformations, and therefore the local water diffusion within the segments in the *z* direction is higher. As the strain increases to S5 and S6, more deformed areas appear, and the local densities at different segments become much higher than those at S1 such that there is not enough space for water diffusion, and the diffusion coefficients in the *z* direction decrease. For the case of 123 water molecules, the number of water molecules is sufficient to form a water chain within the (5,5) CNC. In Fig. 3, one can see that *D*_*z*_ for 123 water molecules significantly increases from 0.047 × 10^−9^ to 0.417 × 10^−9^ m^2^/s (about an 8.9-fold increase) at strains from S1 to S4. Once the deformed areas appear, *D*_*z*_ values will decrease significantly with increasing strain. For the case of 179 water molecules, the deformed areas appear at S6, shown in Fig. 4c, but *D*_*z*_ begins to decrease when strain is larger than S4. Several slightly twisted areas at the (5,5) CNT segments can be seen in Fig. 4c, which makes the cross sections slightly narrower than those at strain 4, leading to more water molecules being blocked at the vertex parts at strain S5.

**Fig. 5:**
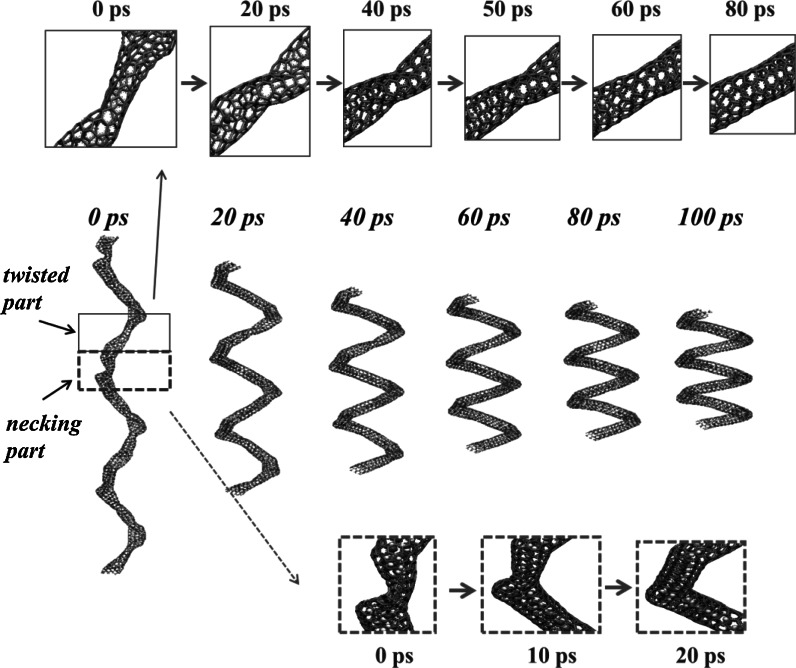
Six *snapshots* at sequential time steps showing local structural variations at one twisted and one necking part deformation in (5,5) CNC

In order to determine whether the deformed (5,5) CNC at higher strain can recover its intact structure at strain of 0 (or stress 0 in the tensile direction), the case of 72 water molecule (5,5) CNC structure at strain S6 is used in the isobaric–isothermal ensemble (NPT). The Parrinello algorithm (Parrinello and Rahman 1981) is used to relax the stress at strain S6 to 1 atm, and the stresses in the *x* and *y* dimensions are set to 0 during the simulation. Six snapshots at sequential time steps are illustrated in Fig. 5, and it is clear that the deformed (5,5) CNC will recover the S1 structure, and the water molecules can flow throughout the (5,5) CNC again. A closer observation of the deformed parts at 0 ps shows that there are two different deformation types for the (5,5) CNC: twisted and necking parts. The twisted parts occur at the (5,5) CNT segments while the necking parts appear at the vertex. The local structural variations for one twisted and one necking part deformation (5,5) CNC are also shown and detailed in Fig. 5 and its insets. The twisted part completely recovers to the (5,5) CNT segment after 80 ps, and the necking part completely recovers to the vertex part at strain S1. This reveals that the (5,5) CNC can repeatedly be used as a water molecule switch by the generation of deformed areas under tensile strain and subsequent structural recovery from the relaxation of tensile strain.

## Conclusion

**Fig. 6:**
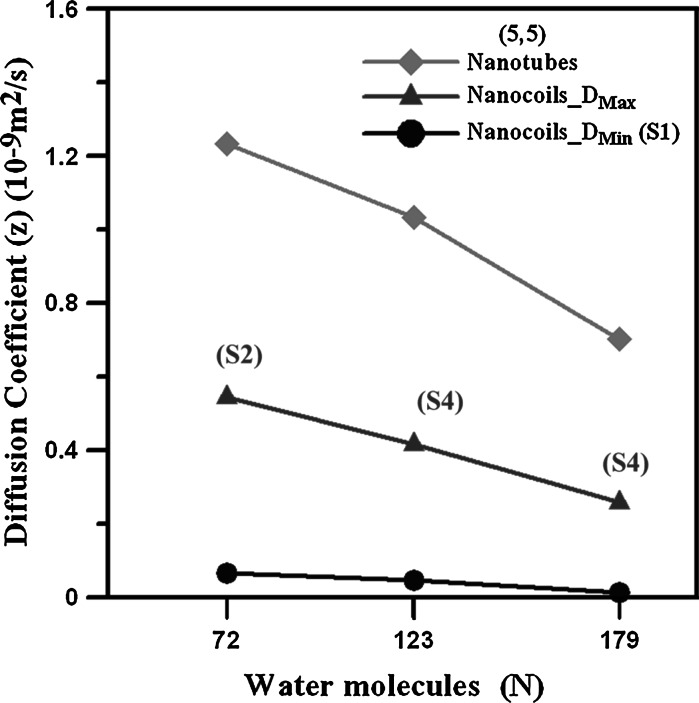
*D*_*z*_ values for systems of different numbers of water molecules within the (5,5) CNT and (5,5) CNC

This study examines the diffusion behaviors of water molecules within the (5,5) CNC at different strains by MD simulation. The diffusion in the axial direction can be enhanced by the tensile strain, and the water molecule flow can be blocked at higher strain, after the deformed areas appear. Figure 6 summarizes the *D*_*z*_ values for systems of different numbers of water molecules within the (5,5) CNT and (5,5) CNC. *D*_Max_ is the largest *D*_*z*_ without blockage, and *D*_Min_ is the lowest value at strain S1 for each case. By applying the tensile strain, the *D*_*z*_ value can be enhanced from 0.065 × 10^−9^ to 0.545 × 10^−9^ m^2^/s for the case of 72 water molecules and increased from 0.014 × 10^−9^ to 0.257 × 10^−9^ m^2^/s for 179 water molecules. For the (5,5) CNT, although the water diffusion in the axial direction is faster than that within the (5,5) CNC, the adjustment of diffusion behavior by axial strain is not significant. As shown in this study, CNCs could be a promising material for the use as an artificial gated bio-channel. Due to their ability to control the flow amount of molecules from one end to the other end of CNCs by axial strain, the applications of CNCs on the nanosyringe, drug nanodelivery, or nanoswitch could be implemented in the future.
